# Potential for transmission of naturally mutated H10N1 avian influenza virus to mammalian hosts and causing severe pulmonary disease

**DOI:** 10.3389/fmicb.2023.1256090

**Published:** 2023-09-12

**Authors:** Mark Zanin, Tran Bac Le, Woonsung Na, Jung-Ah Kang, Hyung-Jun Kwon, Jaehyun Hwang, Eul Hae Ga, Sook-San Wong, Hae-Jin Cho, Daesub Song, Hye Kwon Kim, Dae Gwin Jeong, Sun-Woo Yoon

**Affiliations:** ^1^School of Public Health, The University of Hong Kong, Hong Kong, Hong Kong SAR, China; ^2^Korea Research Institute of Bioscience and Biotechnology, Daejeon, Republic of Korea; ^3^College of Veterinary Medicine, Chonnam National University, Gwangju, Republic of Korea; ^4^Korea Institute of Environment Ecology, Daejeon, Republic of Korea; ^5^College of Veterinary Medicine, Seoul National University, Seoul, Republic of Korea; ^6^Department of Microbiology, College of Natural Sciences, Chungbuk National University, Cheongju, Republic of Korea; ^7^Department of Vaccine Biotechnology, College of Life Sciences and Health Welfare, Andong National University, Andong, Republic of Korea

**Keywords:** avian influenza, H10N1, wild bird, zoonosis, transmission, ferret model

## Abstract

Subtype H10 avian influenza viruses (AIV) are distributed worldwide in wild aquatic birds, and can infect humans and several other mammalian species. In the present study, we investigated the naturally mutated PB2 gene in A/aquatic bird/South Korea/SW1/2018 (A/SW1/18, H10N1), isolated from wild birds during the 2018–2019 winter season. This virus was originally found in South Korea, and is similar to isolates from mainland China and Mongolia. It had low pathogenicity, lacked a multi-basic cleavage site, and showed a binding preference for α2,3-linked sialic acids. However, it can infect mice, causing severe disease and lung pathology. SW1 was also transmitted by direct contact in ferrets, and replicated in the respiratory tract tissue, with no evidence of extrapulmonary spread. The pathogenicity and transmissibility of SW1 in mouse and ferret models were similar to those of the pandemic strain A/California/04/2009 (A/CA/04, H1N1). These factors suggest that subtype H10 AIVs have zoonotic potential and may transmit from human to human, thereby posing a potential threat to public health. Therefore, the study highlights the urgent need for closer monitoring of subtype H10 AIVs through continued surveillance of wild aquatic birds.

## Introduction

Subtype H10 avian influenza A viruses (AIVs) are distributed worldwide in waterfowl (Vijaykrishna et al., 2013), but are also often found in mammalian hosts, including pigs (Wang et al., 2012), minks (Englund, 2000), harbor seals (Krog et al., 2015), and dogs (Su et al., 2014). Human cases were reported in Egypt Pan American Health Organization (2004), Australia in 2010 (Arzey et al., 2012) and China in 2013 (Ma et al., 2015). A fatal human case associated with H10N8 infection in an elderly individual in China was reported Chen et al. (2014). These cases were associated with a history of contact with live poultry, directly or through markets, and there have been no reports of sustained human-to-human transmission to date. Serosurveys in the US and Australia have revealed further instances of exposure of poultry workers to these viruses, which may have led to mild or asymptomatic infections.

The widespread circulation of H10 AIVs, coupled with their ability to infect multiple mammalian species, including humans, makes the zoonotic potential of H10 AIVs a cause of concern. Furthermore, their co-circulation with other AIVs raises the possibility of reassortment, which has already been confirmed with the isolation of an H10N8 AIV in poultry that contained an internal gene cassette from H9N2 AIVs (Liu et al., 2015). Despite their lack of multi-basic cleavage sites and low pathogenicity in general (Kim et al., 2012; El-Shesheny et al., 2018), H10 AIVs are highly pathogenic in chickens, according to *in vivo* tests (Zhang et al., 2016). H10 AIVs are also able to replicate and cause weight loss in mammalian hosts, such as mice (El-Shesheny et al., 2018) and ferrets (Sutton et al., 2017).

H10 AIVs are currently divided into two lineages: Eurasian and North American (Wu et al., 2019). In the present study, we describe the virological and pathological properties of the Eurasian lineage A/SW1/18. This virus was isolated during active surveillance of wild birds in the winter season from 2018 to 2019 in South Korea, and was found to be related to AIVs isolated in mainland China, Mongolia, and Korea. Although it has low pathogenicity and shows a binding preference for avian-type α2,3-linked sialic acids, A/SW1/18 is pathogenic in mice, causing a lethal infection in all exposed animals, and is transmissible via direct contact in a ferret model. Therefore, A/SW1/18 and potentially other H10 AIVs, pose the threat of zoonosis, with the possibility of human-to-human transmission.

## Materials and methods

### Virus isolation and sequencing

The A/SW1/18 virus was isolated from the feces of migratory wild birds in Chungnam province, South Korea (GPS 36.79626, 126.97746) during the 2018–2019 winter season. All collected samples were placed in a transport medium (Noble Bioscience, Republic of Korea) as a 10% suspension and transported to the biosafety level 2 (BL2) lab for further analysis. The A/SW1/18 virus was grown in specific-pathogen-free (SPF) 9-day-old embryonated chicken eggs. To examine the pathogenic properties of A/SW1/18, we generated a recombinant A/CA/04 virus as a positive control using a reverse genetic system (Koo et al., 2018). For molecular characterization, eight genome sequences were prepared and analyzed as previously described (Hoffmann et al., 2001). The sequences were assembled using CLC Sequence Viewer software, version 6.7. Non-coding regions containing the primer sequences were trimmed. Phylogenetic analysis based on each gene sequence was conducted using Molecular Evolutionary Genetics Analysis software (MEGA, version 7.0). Evolutionary distances were computed using the maximum composite likelihood method with 1,000 replicates. Input nucleotide sequences included both the new isolate and reference sequences from the open access resources of the GenBank database for the influenza virus.

### Viral growth kinetics

To evaluate the multistep growth kinetics of A/SW1/18 and A/CA/04 viruses *in vitro*, MDCK, chicken embryonic fibroblasts (DF-1), and primary NHBE cells were infected with the virus at a multiplicity of infection (MOI) of 0.01 plaque-forming units per cell. After 1 h incubation, the virus inoculum was removed, the cells were washed with phosphate-buffered saline (PBS), and infection medium containing 1 μg of tosylsulfonyl phenylalanyl chloromethyl ketone-treated trypsin per ml was added. Supernatants were collected at 12, 24, 36, 48, and 72 h post-infection. The virus titer of each supernatant was determined using a TCID_50_ assay in MDCK cells.

### Receptor binding assay

The binding preference of A/SW1/18 for avian- and human-specific virus receptors was confirmed using a solid-phase direct binding assay, as described previously (Koo et al., 2018). In brief, 10 μg of fetuin (Sigma-Aldrich, MO, USA) was coated and incubated overnight at 4°C and blocked with 5% bovine serum albumin (BSA) in PBS at room temperature for 1 h. After blocking, the plates were washed four times with PBS and incubated with 64 hemagglutinating units (HAU) of each virus at 4°C overnight. After virus removal, the plate was washed as described above and incubated with 0.1 ml of each biotinylated glycan per well at different concentrations at 4°C for 3 h. Glycan binding was detected by adding horseradish peroxidase-conjugated streptavidin (Invitrogen, Carlsbad, CA, USA) followed by 3,3,5,5-tetramethylbenzidine substrate (Sigma-Aldrich), and the optical density was measured at 450 nm using a Synergy HTX multi-mode microplate reader (BioTek, VT, USA). A low-pathogenic H5N2 avian influenza virus, A/Aquatic Bird/Korea/CN2/2009 (A/CN2/09), with a binding preference for the avian receptor, was used as a control (Koo et al., 2018).

### Mouse experiments

For pathogenicity in a mammalian host, 6-week-old female C57BL/6J mice (Koatech, Republic of Korea) were anesthetized with Avertin (Sigma) and inoculated (*n* = 20) intranasally with 10^5^ TCID_50_/30 μl. After inoculation, the mice were checked for body weight loss daily for 2 weeks. The mice were euthanized if they lost more than 30% of their initial body weight. To evaluate viral tissue tropism, inoculated mice were sacrificed (*n* = 12) at 5, 7, and 9 DPI, and their brains, spleens, livers, kidneys, hearts, and lungs were harvested. The tissues were homogenized, and the homogenate supernatant was titrated using a TCID_50_ assay for virus detection. The limit of virus detection was 1 log_10_ TCID_50_/ml. For histopathological analysis, lung tissue samples were fixed in 10% phosphate-buffered formalin and embedded in paraffin and sections (approximately 4–5 μm in thickness) were stained with hematoxylin and eosin (H&E) monoclonal antibody for influenza A virus N protein (ab20343, Abcam, Cambridge, UK), respectively. All images were captured using a Leica DFC 5,400 digital camera and processed using Leica Application Suite v.4.13 (Leica Microsystems, Wetzlar, Germany). All mouse experiments were conducted at the Korea Research Institute of Bioscience and Biotechnology (KRIBB), and were approved by and conducted in accordance with the guidelines of the Institutional Animal Care and Use Committee (IACUC, approval number KRIBB-AEC-16168) of KRIBB.

### Ferret experiments

To assess transmissibility in a mammalian host, 15-to 16-week-old female ferrets (*Mustella putorios furo*) (I. D. Bio Corporation, Republic of Korea) that were serologically negative for seasonal influenza viruses were used. Three ferrets were anesthetized and inoculated intranasally with 10^5^TCID TCID_50_/ml of A/SW1/18 or A/CA/04 viruses in 1 ml of sterile phosphate-buffered saline (PBS, pH 7.4). For direct contact transmission experiments, ferrets housed in a cage placed inside an isolator were intranasally inoculated with 10^5^ TCID_50_/ml of the test virus. 24 h later, three naïve ferrets were introduced into the same cage and naive ferrets were added to the other half of the cage, which was separated by double layers of wire mesh allowing only respiratory droplet (RD) contact. Nasal washes were collected daily, beginning at 2 DPI (i.e., 1 d after contact). To evaluate viral tissue tropism, inoculated ferrets were sacrificed at 5 DPI, and their brains, spleens, livers, kidneys, hearts, and lungs were harvested. The tissues were homogenized, and the homogenate supernatant was titrated using a TCID_50_ assay for virus detection. The limit of virus detection was 1 log_10_ TCID_50_/ml. All contact ferrets were euthanized at 21 dpi, and their blood samples were tested for specific antibodies to a homologous virus using a hemagglutination inhibition assay. For pathological analysis, lung tissue samples were fixed in 10% phosphate-buffered formalin and embedded in paraffin, and sections (approximately 4–5 μm in thickness) were stained with H&E and monoclonal antibody for influenza A virus N protein (ab20343, Abcam, Cambridge, UK), respectively. All images were captured using a Leica DFC 5,400 digital camera and processed using Leica Application Suite v.4.13 (Leica Microsystems, Wetzlar, Germany). For All ferret experiments were conducted at KRIBB and Chonnam National University (CNU, Republic of Korea), and were approved by and conducted in accordance with the guidelines of the IACUC (approval number KRIBB-ACE-19203 and CNU-IACUC-YB-R-2021-144) of KRIBB.

### Statistical analysis

Statistically significant differences in multistep experimental groups were determined using analysis of variance (ANOVA) in GraphPad Prism version 5.03.

## Results

### Genetic relatedness of A/SW1/18 AIV

**FIGURE 1:**
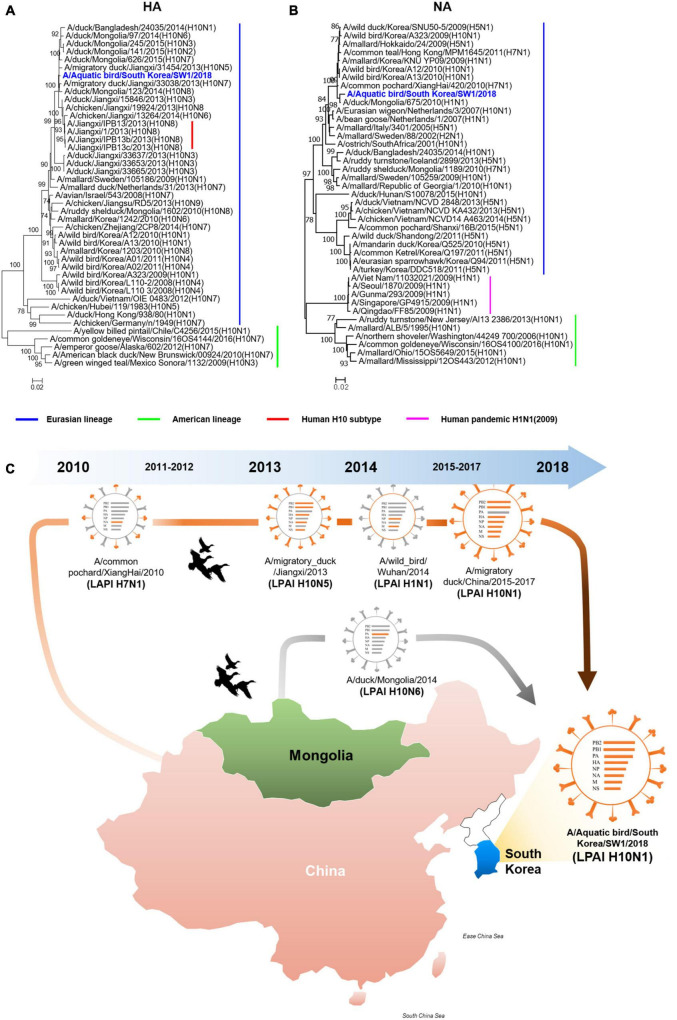
The hemagglutinin and neuraminidase of A/aquatic bird/Republic of Korea/A/SW1/18/2018 were of Eurasian lineage. Hemagglutinin **(A)** clustered with subtype H10 avian influenza viruses isolated in Mongolia, China and Korea. The neuraminidase **(B)** clustered with H5 and H10 avian influenza viruses isolated in Mongolia, China and Korea in 2009 to 2010. The evolutionary distances were computed using the Maximum Composite Likelihood method with 1,000 replicates. The input nucleotide sequences included both new isolate and reference sequences obtained from the Influenza Virus Resource at the National Centre for Biotechnology Information (NCBI). The statistic values greater than 70% a measure of reliability from a bootstrap (*n* = 1,000) iterations were showed. Putative generation of A/aquatic bird/South Korea/A/SW1/18/2018 has origins in aquatic bird viruses from mainland China and Mongolia. Each color represents an individual virus background and genes from top to bottom are PB2, PB1, PA, HA, NP, NA, M, and NS **(C)**. The geographic map was built using ggmap and ggplot2 packages on R version 4.3.1.

The A/SW1/18 was isolated from a wild bird fecal specimen in Chungnam Province, South Korea, during the 2018–2019 winter season. Hemagglutinin (HA) from A/SW1/18 clustered with that of other subtype H10 AIVs isolated in Mongolia, China, and Korea from 2013 to 2015, whereas A/SW1/18 neuraminidase (NA) clustered with that of H5 and H10 AIVs isolated in Mongolia, China, and Korea during 2009–2010 (Figures 1A, B). The internal genes of A/SW1/18 were also of Eurasian lineage, and were the more closely related to AIVs isolated from aquatic birds in Mongolia, China, and South Korea (Supplementary Figure 1). A/SW1/18 is composed of gene segments that could be traced to wild bird viruses in mainland China and Mongolia (Figure 1C). The amino acid (aa) sequences of the HA proteins of A/SW1/18 did not contain a polybasic amino acid series (RXR/KR↓G or RR/KXR↓G in H5Nx HPAI) (Kido et al., 2008), but only a single amino acid at the HA cleavage site (PELMQGR↓GLF), which is the reason for the low pathogenicity of avian influenza viruses in chickens (Zhang et al., 2016). A/SW1/18 preferentially binds to avian-like receptors with no amino acid substitutions at positions 186, 222, and 224 (G to S) in HA, which are well-recognized as human-like α2-6-linked receptors (Liu et al., 2015). No amino acid substitutions associated with resistance to NA inhibitors were observed. However, a single amino acid substitution at position 627 of glutamine to lysine (E627K) in PB2 of A/SW1/18 was identified, and is a critical determinant of virulence in mammalian hosts (Herfst et al., 2012).

### Replication kinetics of A/SW1/18 AIV

**FIGURE 2:**
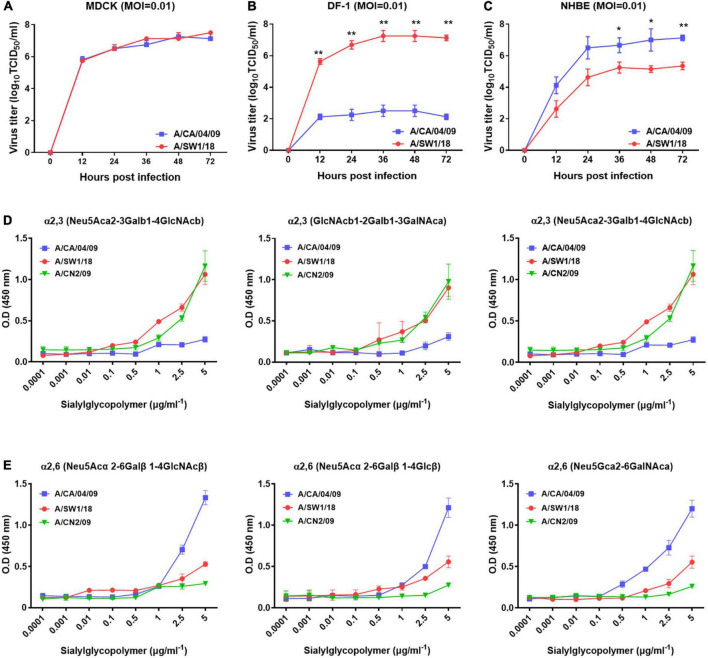
A/aquatic bird/South Korea/A/SW1/18/2018 (A/SW1/18) replicated in avian and mammalian cells. Replication kinetics and peak viral titers of A/SW1/18 and the pandemic strain A/California/04/2009 (A/CA/04) were similar in MDCK cells **(A)**. A/SW1/18 showed significantly greater replication kinetics and peak viral titers compared to A/CA/04 in DF-1 cells **(B)**. A/SW1/18 replicated in NHBE cells but at slower kinetics and to lower peak titers compared to A/CA/04 **(C)**. Graphs show the mean ± standard error of the mean of three individual experiments. *p < 0.05 and **p < 0.001. A/aquatic bird/South Korea/A/SW1/18/2018 (A/SW1/18) showed a binding preference for α2,3-linked sialic acids. A/SW1/18 and the avian influenza virus A/aquatic bird/South Korea/CN2/2009 (A/CN2) bound with significantly higher affinity to α2,3(Neu5Aca2-3Galb1-4GlcNAcb), α2,3(GlcNAcb1-2Galb1-3GalNAca) and α2,3(Neu5Aca2-3GalNAca) sialoglycopolymers compared to the pandemic strain A/California/04/2009 (A/CA/04) **(D)**, respectively. The affinity of A/SW1/18 and A/CN2 for these α2,3-linked sialic acids was similar. A/CA/04 showed significantly greater affinity for α2,6 (Neu5Acα 2-6Galβ1-4GlcNAcβ), α2,6(Neu5Acα2-6Galβ1-4Glcβ), and α2,6(Neu5Gcα2-6GalNAca) sialoglycopolymers compared to A/SW1/18 and A/CN2 **(E)**, respectively.

In MDCK cells, the replication of A/SW1/18 (peak titer 7.5 ± 0.5) was similar to that of the A/CA/04 (peak titer 7.25 ± 0.25; Figure 2A). In NHBE cells, A/SW1/18 replicated to a robust peak titer of 5.3 ± 0.35, although this was significantly less than that of CA/04 at 7.25 ± 1.25 (Figure 2B). In the chicken fibroblast cell line DF-1, A/SW1/18 replicated to a significantly higher peak titer of 7.25 ± 1.25 compared to that of A/CA/04 (Figure 2C), demonstrating that, unlike A/CA/04, A/SW1/18 is capable of robust replication in mammalian and avian cells *in vitro*.

### Receptor binding preference of A/SW1/18 AIV

As A/SW1/18 is capable of replication in avian and mammalian cells, we studied the sialic receptor binding preferences of this virus in comparison to that of A/CA/04 and A/CN2/09, which are known to have a binding preference for the avian receptor. The pandemic virus A/CA/04 bound relatively weakly to α2,3-linked sialic acids, while A/SW1/18 and a/CN2/09 bound with similar affinities that were significantly greater than that of A/CA/04 (Figure 2D). Conversely, A/SW1/18 and A/CN2/09 bound with significantly lower affinity to the α2,6-linked sialic acids than CA/04 (Figure 2E).

### Pathology of A/SW1/18 AIV in a mouse model

**FIGURE 3:**
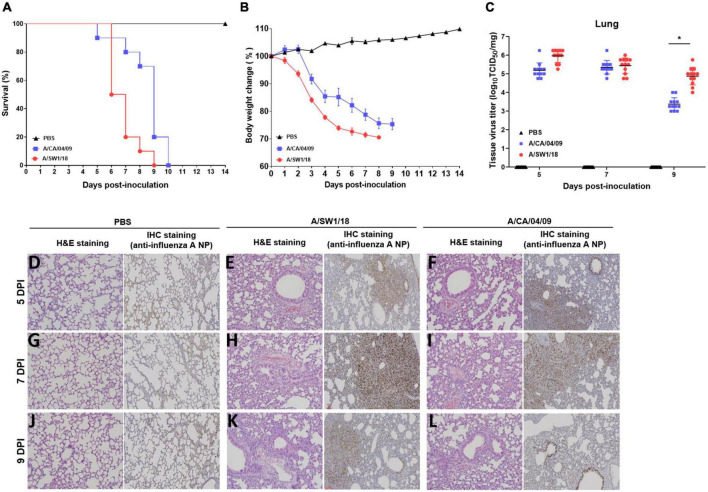
A/aquatic bird/South Korea/A/SW1/18/2018 (A/SW1/18) caused severe disease in mice. Inoculation with A/SW1/18 and A/California/04/2009 (A/CA/04) led to 100% mortality by 9- and 10-days post inoculation (DPI) in C57BL/6 mice, respectively **(A)**. A/SW1/18 was associated with significantly more rapid mortality and weight loss compared to A/CA/04 **(B)**. Viral titers measured in the lungs of mice inoculated with A/SW1/18 or A/CA/04 were similar at five and seven DPI. At nine DPI, lung titers of A/SW1/18 were significantly greater compared to A/CA/04 **(C)**. Lung pathology in PBS- **(D,G,J)**, A/SW1/18- **(E,H,K)**, and A/CA/04-inoculated **(F,I,L)** mice was characterized by hematoxylin and eosin staining and immunohistochemical staining with monoclonal antibody of influenza A virus N protein. Images are representative of *n* = 20 mice. Data points in **(A)** and **(B)** represent the mean ± standard error of the mean of *n* = 20 mice. Bars in **(C)** represent the mean ± standard error of the mean of *n* = 12 mice. Magnification × 100. *p < 0.05.

Since the substitution of E627K in the PB2 gene contributes to increased virulence and adaptation in mammalian hosts, we evaluated the biological characteristics and pathogenicity of A/SW1/18 in mammalian hosts. Interestingly, A/SW1/18 caused 100% mortality in a C57BL/6 mouse model, similar to A/CA/04 (Figure 3A). However, mice inoculated with A/SW1/18 showed a more rapid progression of disease, as evidenced by a significant difference in the period of survival and percentage loss in body weight between the two groups (Figures 3A, B). Lung viral titers were similar in mice inoculated with A/SW1/18 or A/CA/04 at 5- and 7-days post-inoculation (DPI). At 9 DPI, viral titers were significantly greater in A/SW1/18-inoculated mice (peak titer 4.87 ± 0.47) than in A/CA/04-inoculated mice (peak titer 3.37 ± 0.34; Figure 3C). Lung pathology at 5, 7, and 9 DPI indicated that both viruses caused severe disease. Expansion of inflammation and alveolar collapse, cell debris in bronchiolar lumen, and lung severe collapse were observed at 7 and 9 dpi in virus-inoculated mice. Also, lung viral replication was confirmed similar in mice inoculated with A/SW1/18 or A/CA/04 by immunohistochemistry against viral nucleoprotein (Figures 3D–L).

### Transmissibility of A/SW1/18 AIV in a ferret model

**FIGURE 4:**
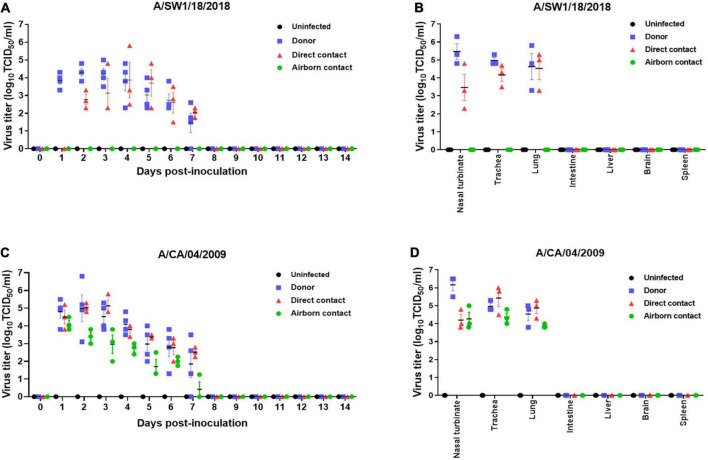
A/aquatic bird/South Korea/A/SW1/18/2018 (A/SW1/18) was transmissible by direct contact in the ferret model and replicated in tissues of the respiratory tract. Viral titers were detected in nasal washes obtained from donor ferrets inoculated with A/SW1/18 **(A)** or A/California/04/2009 (A/CA/04) **(C)** collected one to 7 days post inoculation (DPI). In direct contact ferrets, viral titers were detected in nasal washes collected two to seven DPI in the A/SW1/18 groups and one to seven DPI in the A/CA/04 group **(A,C)**, respectively. Viral titers were not detected in nasal washes obtained from uninfected ferrets **(A,C)**. Viral titers were detected in the nasal turbinates, trachea and lungs of donor and direct contact ferrets in the A/SW1/18 **(A)** and A/CA/04 **(D)** groups. Virus titers in nasal turbinates were significantly greater in donor ferrets compared to direct contact ferrets in A/SW1/18 and A/CA/04 groups **(B,D)**. Viral titers were not detected in the intestines, liver, brain or spleen of any ferrets **(B,D)**. Graphs represent the mean ± standard error of the mean of *n* = 3 ferrets.

**FIGURE 5:**
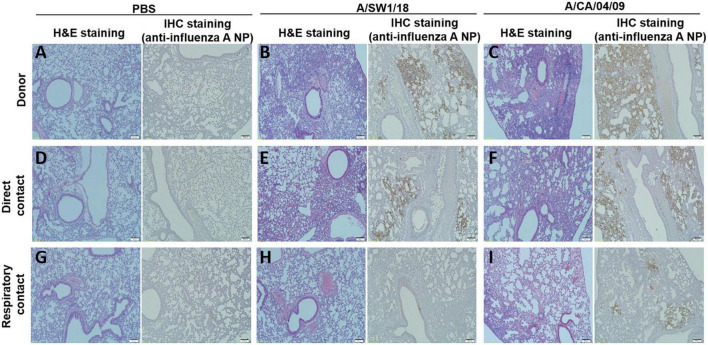
Lung pathology was of greater severity in donor and direct contact ferrets in the A/aquatic bird/South Korea/A/SW1/18/2018 (A/SW1/18) group compared to the A/California/04/2009 (A/CA/04) group. Compared to direct contact ferrets in the PBS groups **(A,D)**, lung pathology in the A/SW1/18 groups **(B,E)** was more severe, characterized by hematoxylin and eosin staining and immunohistochemical staining with monoclonal antibody of influenza A virus N protein. Pathology in the A/CA/04 groups **(C,F)** was more severe compared to the PBS groups but less severe compared to the A/SW1/18 groups and was characterized by hematoxylin and eosin staining. Compared to respiratory contact ferrets, lung pathology in the A/CA/04 groups **(I)** was only severe compared to the PBS **(G)** and A/SW1/18 groups **(H)** groups, characterized by hematoxylin and eosin staining and immunohistochemical staining with monoclonal antibody of influenza A virus N protein. Images are representative of *n* = 3 ferrets. Magnification × 100.

The pathogenicity of A/SW1/18 in a mouse model led us to determine whether this virus was also capable of spread between mammals. To address mammalian transmission, we used a ferret model to determine the transmissibility of A/SW1/18, and compared it to that of A/CA/04, which shows robust replication and transmission in ferrets (Yoon et al., 2015). A/SW1/18 was capable of replicating in the respiratory tract of ferrets, and was transmitted between animals through direct contact; however, its RD transmissibility in ferrets is limited. CA/04 exhibited more efficient transmission by direct and RD-contact than A/SW1/18 (Figures 4A, C). A/SW1/18 was detected in the nasal washes of animals in direct contact with infected animals at 2 DPI, whereas A/CA/04 was detected at 1 DPI (Figure 4A), with A/CA/04 detected at titers approximately one log greater than those of A/SW1/18 at all-time points (Figure 4C). Ferrets infected with either virus showed obvious clinical signs, such as coughing and wheezing, but there were no obvious differences in the signs observed in ferrets inoculated with the different viruses. A/SW1/18 replicated in the nasal turbinates, trachea, and lungs of inoculated and direct-contact ferrets (Figure 4B). A/CA/04 and A/SW1/18 replicated with similar titers in the nasal turbinates, trachea, and lungs. There was no evidence of extrapulmonary spread of A/CA/04 or A/SW1/18, as titers were not detected in the intestine, liver, brain, or spleen (Figures 4B, D). In ferrets infected with A/CA/04, lung pathology signaling disease was greater in donor ferrets than in direct-contact ferrets and RD-contact ferrets, characterized by the viral pathology of the lung tissue. Surprisingly, when compared to A/CA/04, A/SW1/18 was associated with a greater lung pathology in both donor and direct-contact ferrets. Pulmonary parenchyma consolidation infiltration and alveolar septal infiltration observed as well as interstitial pneumonia manifested by alveolitis (Figure 5). Homologous antibody titers showed that all the serum samples collected from A/CA/04 infected and RD-contacted ferrets were reactive to homologous virus at 21 dpi. However, only A/SW1/18 -infected contact ferrets seroconverted at the end of the experiment (Supplementary Table 1).

## Discussion

Subtype H10 AIVs have been reported worldwide in wild birds and poultry. In South Korea, several H10 subtype AIVs were isolated from wild birds during the winter between July 2008 and July 2011, but there is a lack of surveillance and available data on their biological characteristics. In the present study, we showed that an H10N1 AIV isolated from wild birds can cause severe infections and transmit in mammalian models, even while displaying low pathogenicity and a binding preference for avian-like α2,3-linked sialic acids. However, the specific amino acid substitution of PB2 E627K has not been previously reported in other H10 AIVs in South Korea since 2008. The present study revealed that A/SW1/18 contains E627K in PB2, which is associated with viral replication and pathogenicity in the respiratory tract of mammalian hosts. Our study indicates that H10 AIVs pose a threat to further zoonotic events and potential human-to-human transmission.

All human cases of H10N8 infection in China in 2013 were associated with a history of visits to live poultry markets. This virus contains internal genes originating from H9N2 viruses that are enzootic in poultry in China. The co-circulation of H10 AIVs with subtypes such as H9N2 carries the risk of emergence of new strains with zoonotic potential, which can cause outbreaks in humans. This is how the H7N9 strain emerged, which caused multiple waves of outbreaks in China in 2013 in China. H7N9 arose from reassortment events that involved the internal genes of the H9N2 virus. The circulation of these viruses in aquatic birds means that the chances of human contact with them is low. However, if they exist in poultry, the chances of transmission to humans are greatly increased, particularly in the context of live poultry markets and backyard farms. The incidence of such transmission has been confirmed by the detection of seropositive poultry workers in serosurveys in the US and Australia.

In the present study, we used two animal models for influenza virus infection, in mice and ferrets. The mouse model has been used extensively to gain insights into the mammalian pathogenicity of AIVs (Koçer et al., 2012; Zanin et al., 2017). These studies used the DBA2/J mouse strain, which is relatively sensitive to IAV infection, thus making it a useful screening tool for mammalian pathogenicity. A study of four H10 AIVs isolated in Bangladesh in DBA2/J mice revealed varying pathogenicity, with mortalities ranging from 0 to 100%, although all viruses replicated and caused weight loss (El-Shesheny et al., 2018). In the present study, we used the C57BL/6 mouse strain, which is relatively resistant to AIVs, and confirmed that A/SW1/18 was capable of causing severe disease on par with the pandemic strain A/CA/04. Based on these experiments in mice, we speculate that host-though resistance to AIVs in general allows increased viral replication of A/SW1/18, which subsequently induces more tissue damage, resulting in the death of the host.

H10 AIV inoculation in ferrets leads to replication and seroconversion (Sutton et al., 2017) however, the transmissibility of H10 AIVs in this model remains unclear. Our data showed that A/SW1/18 was capable of replicating in the respiratory tract tissue of ferrets, and that transmission by direct contact was possible. In comparison with A/CA/04, which is capable of being transmitted in ferrets both by direct contact and via the airborne route, A/SW1/18 replicated to similar titers in the respiratory tissue, albeit detected 1 day later in A/SW1/18-inoculated ferrets and their contacts. Interestingly, unlike A/CA/04, which has a strong binding preference for mammalian-like α2,6-linked sialic acids, A/SW1/18 bound relatively weakly, showing a strong preference for avian-like α2,3-linked sialic acids. The naturally substituted PB2 E627K in A/SW1/18 contributed to the efficient viral replication of H10 AIVs in the human upper respiratory tract, and based on these ferret experiments, it can be inferred that subsequent transmissions could be effective as well. These data indicate that A/SW1/18, and perhaps other similar low-pathogenic H10 AIVs, pose a potential threat of human-to-human transmission. Although, A/SW1/18 did not impact the binding preference for mammalian-like sialic acids, naturally PB2 gene through substitution of E627K was sufficient to enhance the polymerase activity in mammalian host and subsequently increased viral virulence in mice and ferret animal model.

In summary, the present study revealed that the Eurasian H10N1 AIV circulating in aquatic birds in South Korea is related to other aquatic bird viruses isolated in mainland China, Mongolia, and South Korea. This low-pathogenic virus is capable of causing severe disease in mice and transmission via direct contact in ferrets without prior adaptation. These data reveal that H10 AIVs are a concern to human health, and should be monitored closely through continued surveillance of wild aquatic birds.

## Data availability statement

The datasets presented in this study can be found in online repositories. The names of the repository/repositories and accession number(s) can be found in this article/Supplementary material.

## Ethics statement

The animal study was approved by the Korea Research Institute of Bioscience and Biotechnology. The study was conducted in accordance with the local legislation and institutional requirements.

## Author contributions

S-WY: Conceptualization, Investigation, Supervision, Writing—original draft, Writing—review and editing. MZ: Project administration, Writing—original draft. TL: Investigation, Methodology, Writing—original draft. WN: Investigation, Methodology, Writing—original draft. J-AK: Data curation, Formal Analysis, Writing—original draft. H-JK: Data curation, Formal Analysis, Writing—original draft. JH: Data curation, Formal Analysis, Writing—original draft. EG: Data curation, Formal Analysis, Writing—original draft. S-SW: Data curation, Formal Analysis, Writing—original draft. H-JC: Resources, Writing—original draft. DS: Data curation, Formal Analysis, Writing—original draft. HK: Conceptualization, Supervision, Writing—original draft. DJ: Funding acquisition, Writing—original draft.
