# Supplementary material for: Potential for transmission of naturally mutated H10N1 avian influenza virus to mammalian hosts and causing severe pulmonary disease
Source: Front Microbiol. 2023 Sep 12;14:1256090. doi: 10.3389/fmicb.2023.1256090 (PMC10536253; doi:10.3389/fmicb.2023.1256090)
Supplement: Supplementary file 1 [file Data_Sheet_1.docx]

Supplementary Material

**Potential for transmission of naturally mutated H10N1 avian influenza virus to mammalian hosts and causing severe pulmonary disease**

Zanin Mark^1,†^, Tran Bac Le^2,†^, Woonsung Na^3,†^, Jung-Ah Kang^2^, Hyung-Jun Kwon^3^, Jaehyun Hwang^3^, Eul Hae Ga^3^, Sook-San Wong^1^, Hae-Jin Cho^4^, Daesub Song^5^, Hye Kwon Kim^6,^*, Dae Gwin Jeong^2,^*, Sun-Woo Yoon^2,7,^*

*** Correspondence:** Sun-Woo Yoon: [syoon@anu.ac.kr](mailto:syoon@anu.ac.kr), Hye Kwon Kim: [khk1329@chungbuk.ac.kr](mailto:khk1329@chungbuk.ac.kr), Dae Gwin Jeong: [dgjeong@kribb.re.kr](mailto:dgjeong@kribb.re.kr)

# Supplementary Figure

## Phylogenetic analysis

## Phylogenetic analysis based on each gene sequence was conducted using Molecular Evolutionary Genetics Analysis software (MEGA, version 7.0). Evolutionary distances were computed using the maximum composite likelihood method with 1000 replicates. Input nucleotide sequences included both the new isolate and reference sequences from the open access resources of the GenBank database for the influenza virus.

## Seroconversion

## Virus-specific antibodies were measured in sera on day 21 post-inoculation using the Hemagglutination inhibition (HI) assays. For the HI assay, virus-inoculated serum samples were treated with receptor destroying enzyme (RDE, CDC, Atlanta) followed by 30 min heating at 56°C. Every serum sample was serially diluted in 25 μl PBS and then mixed with an equal volume of PBS containing 4 hemagglutinating units (HAU) of respective A/SW1/18 and A/CA/04 virus. Incubated the plates for half an hour at room temperature and added 50 μl of 0.5% chicken red blood cells. The resulting HAI titer was the inverse of the last dilution that inhibited agglutination.

## Supplementary Figure and Table

**
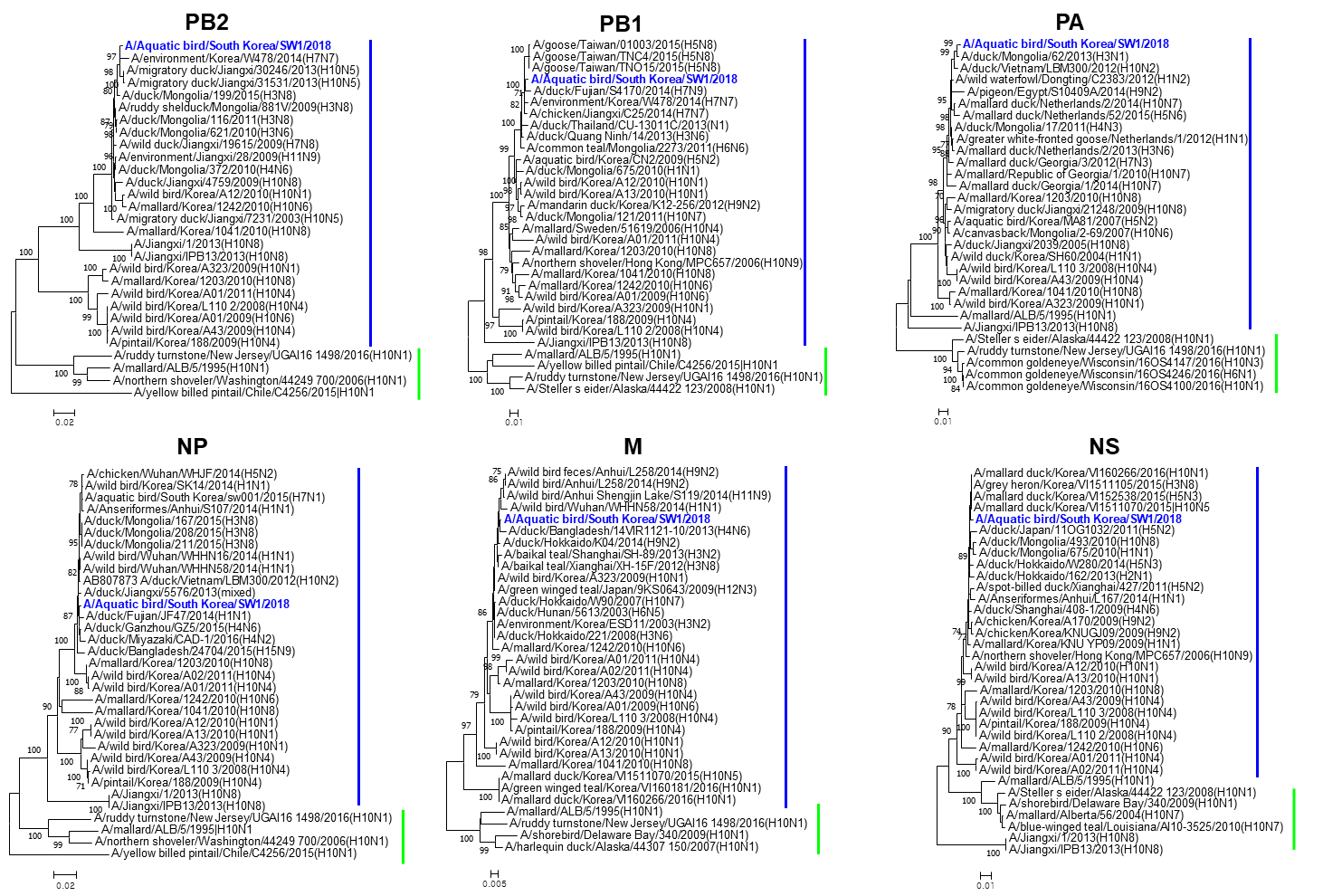
**

**Supplementary Figure 1.** The internal genes of A/aquatic bird/South Korea/A/SW1/18/2018 (H10N1) were of Eurasian lineage. PB2, PB1, PA, NP, M, and NS gene clustered with viruses isolated from aquatic birds in Mongolia, China South Korea. The evolutionary distances were computed using the Maximum Composite Likelihood method with 1000 replicates. The input nucleotide sequences included both new isolate and reference sequences obtained from the Influenza Virus Resource at the National Centre for Biotechnology Information (NCBI). The statistic values greater than 70% a measure of reliability from a bootstrap (n=1000) iterations were showed.

**Supplementary Table 1.** Homologous-antibody titers measured by the hemagglutination inhibition test in a study of transmission in ferrets

|  | **A/SW1/2018** | **A/CA/04/2009** |
| --- | --- | --- |
| Donor ferret | 320, 320, 640 | 640, 1280, 640 |
| Direct-contact ferret | 160, 160, 320 | 320, 320, 320 |
| Respiratory contact ferret | < 10, < 10, < 10 | 160, 320, 160 |
